# Gender Norms and Weight Control Behaviors in U.S. Adolescents: A Prospective Cohort Study (1994–2002)

**DOI:** 10.1016/j.jadohealth.2019.08.020

**Published:** 2020-01

**Authors:** Jason M. Nagata, Benjamin W. Domingue, Gary L. Darmstadt, Ann M. Weber, Valerie Meausoone, Beniamino Cislaghi, Holly B. Shakya

**Affiliations:** a Department of Pediatrics, University of California, San Francisco, San Francisco, California; b Graduate School of Education, Stanford University, Stanford, California; c Department of Pediatrics, Stanford University School of Medicine, Stanford, California; d School of Community Health Sciences, University of Nevada, Reno, Nevada; e Stanford Center for Population Health Sciences, Stanford University, Stanford, California; f Department of Global Health and Development, London School of Hygiene and Tropical Medicine, London, United Kingdom; g Division of Infectious Disease and Global Public Health, University of California, San Diego, La Jolla, California

**Keywords:** Gender norms, Adolescent health, Eating behaviors, Body image, Weight control behaviors, Dieting

## Abstract

**Purpose:**

The aim of this article was to determine the relationship between gender norms and weight control behaviors in U.S. adolescents.

**Methods:**

We analyzed prospective cohort data from the National Longitudinal Study of Adolescent to Adult Health (N = 9,861), at baseline in 1994–1995 (ages 11–18 years, Wave I), 1-year follow-up (ages 12–19 years, Wave II), and 7-year follow-up (ages 18–26 years, Wave III). The primary exposure variable was a measure of one's gender normativity based on the degree to which males and females behave in ways that are similar to the behaviors of their same-gender peers. The outcome variable was an individual's weight control attempts (trying to lose or gain weight) and behaviors (dieting, fasting/skipping meals, vomiting, or weight-loss pills/laxatives/diuretics to lose weight or ate different/more foods than usual or taking supplements to gain weight).

**Results:**

In logistic regression analyses controlling for potential confounders, a higher baseline individual gender normativity score (higher femininity in females and higher masculinity in males) was associated with weight loss attempts (β = .10; *p* = .01) and weight loss behaviors (β = .18; *p* < .001) in girls but was associated with weight gain attempts (β = .18; *p* < .001) and behaviors (β = .16; *p* < .001) in boys at 1-year follow-up. Higher individual gender normativity score was protective of weight loss attempts (β = −.15; *p* < .001) and weight loss behaviors (β = −.17; *p* < .001) in males but not females at 7-year follow-up. Loess plots provided visualizations of significant relationships.

**Conclusions:**

Gender norms may reinforce a thinner body ideal for girls but a larger ideal for boys.

**Implications and Contribution**

In this longitudinal cohort study of U.S. adolescents, gender norms were positively associated with weight loss attempts and behaviors for girls and negatively associated for boys. In boys, gender norms were positively associated with weight gain attempts and behaviors.

Gender norms are societies' rules and standards that guide and constrain social behaviors for boys and girls, or men and women, in regard to how they are supposed to act, think, and feel [1]. Gender norms are often perpetuated and maintained by reinforcement or sanctioning in families, schools, communities, and the media [1]. Gender norms may play a role in body image–related ideals and behaviors [2,3]. For instance, the idealized female body in the U.S. is thin, which contributes to body image dissatisfaction among women [4]. Research has shown that even some females who are normal or underweight perceive themselves as overweight [4]. Approximately 60% of adolescent girls in the U.S. report attempts to lose weight, findings that have remained stable from 1999 to 2015 [5,6]. In contrast, the idealized masculine male body image in U.S. popular media and action figures is large and muscular [7]. Nearly one third of adolescent boys in the U.S. report attempts to gain weight or muscle, compared with only 7% of U.S. adolescent girls, findings that have remained relatively stable from 2002 to 2015 [6,8].

Weight control attempts can include disordered behaviors such as vomiting, fasting, and using laxatives, diuretics, and/or other medications to lose weight [9]. Among adolescents, the prevalence of vomiting, fasting, and laxatives remained relatively stable from 1999 to 2013, whereas diet pill use has decreased [10]. These disordered eating behaviors are associated with increased risk for eating disorders [11,12], depression [13], alcohol and tobacco use [14], and poor nutritional intake and quality [13]. Weight gain behaviors can include eating more, consuming supplements, and anabolic-androgenic steroid use [15]. Adolescence is a key time for the development of body image–related concerns and eating disorders [16]. However, little research, to date, has examined the association between adolescent gender norms and weight control behaviors particularly in community-based adolescent samples with longitudinal data (i.e., where gender norms temporally precede weight control behavior outcomes).

Adolescence is also a time of particular sensitivity to norms and acceptance from peers. Although validated measures have been developed to measure conformity to gender norms in adults [1,2], these have not been validated in adolescent populations. Gender norm measures in existing data sets are rare [17]; thus, proxy measures may be generated. A recent study developed a proxy measure for gender norm conformity in adolescence using existing data from the National Longitudinal Study of Adolescent to Adult Health (Add Health) [18], but this has not yet been examined in relation to weight control behaviors, which previous research has suggested is a gendered health outcome [19]. Understanding how gender norms affect adolescent behaviors is important as these may have implications for health outcomes, policy, and program design. The objective of this study was to determine the association between a gender norms scale and weight control attempts and behaviors among U.S. adolescents, by gender. We hypothesized that an increased alignment with gender norms would be associated with weight loss attempts and behaviors in adolescent girls and weight gain or muscle-building attempts and behaviors in adolescent boys.

## Methods

### Study design and sample

We used data from Add Health, a nationally representative cohort of youth in the U.S. that has been followed from adolescence through adulthood [20]. Systematic sampling methods and implicit stratification were applied to the baseline sample to ensure that the high schools and middle schools selected were representative of U.S. schools with respect to region of the country, urbanicity, size, type, and ethnicity. For this particular study, we used the restricted-use baseline sample (Wave I, 1994–1995; age 11–18 years), 1-year follow-up data (Wave II, 1996; age 12–19 years), and 7-year follow-up data (Wave III, 2001–2002; age 18–26 years). Wave IV (2008; age 24–32 years) did not collect data on weight control attempts or behaviors (outcomes); thus, we did not use this Wave. We included subjects in the sample who had data at all three waves (I–III). Of the 18,922 adolescents in the nationally representative weighted baseline sample, 9,861 (52.1%) had completed 1- and 7-year follow-up data and were included (Appendix A). Further details about the Add Health study design, coordinated by the Carolina Population Center, can be found elsewhere [20]. The University of North Carolina Institutional Review Board approved all Add Health study procedures, and the University of California, San Francisco, Institutional Review Board deemed this specific project exempt.

### Procedures

At Add Health's baseline and 1- and 7-year follow-up, an interviewer traveled to the home or another suitable location for the participant. Written consent was obtained from the parent if the participant was aged younger than 18 years or from the participant if aged 18 years or older. Interviews lasted approximately 90 minutes and were conducted in as private an area as possible. Audio computer-assisted self-interview (baseline) and computer-assisted self-interview (follow-up) were used by participants to answer potentially sensitive questions.

### Measures

#### Exposure variable

##### Gender normativity score

The primary exposure variable was a measure of one's gender normativity, which was developed by Cleveland et al. [21], refined by Nowotny et al. [22], and further refined and applied by Fleming et al. [18]. The measurement technique, developed from Add Health measures, assessed the degree to which males and females behave in ways that are predictive of their self-reported sex. The measure is based on a combination of self-reported behaviors and attitudes; it was designed to capture the performance of gender rather than self-reported ideologies or attitudes toward gender-specific social expectations. Fleming et al. [18] provided evidence for the reliability (using split-half reliability) and construct validity (both convergent and divergent validity) of the original measure. We calculated the measure based on the variables identified by Fleming et al. for Wave I (Appendix A) but omitted variables that measured the same characteristics as our outcomes (such as eating, body image, or exercise), as we have done previously [17,23]. The area under the curve for the gender normativity score with and without the potentially endogenous variables were .867 and .859, respectively, at Wave I. These changes will lead to a slight reduction in measurement precision. The resulting effect will be to slightly reduce our power to detect effects, but we are still well-powered with the modified predictor, given the size of the Add Health sample.

The gender normativity score was first calculated at the individual level. For a given school, the survey measures were first trained (via logistic regression) to predict gender using all adolescents in other schools. The regression model coefficient estimates (from the out-of-school adolescents) were then used to compute predicted probabilities of being a male or female for the adolescents within the school, which we refer to as their gender normativity. To capture the potential effects from the school environment, the modified gender normativity score was also aggregated at the school level. To construct the school-level gender normativity score, sex-specific individual scores were aggregated to the mean of same-sex school-level peers using jackknife resampling (i.e., leaves out the focal individual from the mean). Respondents with fewer than five same-sex individuals sampled in their school were dropped from formal tests (<1% of the sample). Additional details of the construction of the gender normativity score have been reported previously [17,23].

A gender normativity of .9 suggests an estimated 90% probability of the person being the binary sex assignment (male or female) they were given in the survey. A high score for girls represents more “feminine,” and a high score for boys represents more “masculine.” Because not all individuals were likely to engage in all the behaviors typical of their gender, the average score on the gender normativity measure was .69.

#### Outcomes variables

##### Weight control attempts

Participants were asked at baseline (Wave I) and follow-up (Waves II and III), “Are you trying to lose weight, gain weight, or stay the same weight?” Response choices included “trying to lose weight,” “trying to gain weight or bulk up,” “trying to stay the same weight,” or “not trying to do anything about your weight.” “Trying to lose weight” was coded as a weight loss attempt. “Trying to gain weight” was coded as a weight gain attempt.

##### Weight loss behaviors

Participants who reported weight loss attempts were then asked, “During the past 7 days, which of the following things did you do in order to lose weight or to keep from gaining weight?” Response choices included (1) dieting, (2) fasted or skipped meals, (3) made yourself throw up, (4) took weight-loss pills, (5) took laxatives, or (6) used diuretics. Those who had affirmative responses to any of 1–6 were coded as engaging in weight loss behaviors. These questions were adapted from validated eating behavior measures used in the Adolescent Health Survey and similar to those used in Project Eating Among Teens (85% agreement ≥1 behavior, r = .76) [24,25], except that the time frame was 7 days instead of 28 days to be consistent with the 7-day time frame of other validated questions in the Add Health survey on nutrition and physical activity.

##### Weight gain behaviors

Participants who reported weight gain attempts were then asked, “During the past 7 days, which of the following things did you do in order to gain weight or bulk up?” Response choices included (1) ate different foods than usual, (2) ate more, or (3) took food supplements. Those who had affirmative responses to any of 1–3 were coded as engaging in weight gain behaviors. These questions were adapted from validated eating behavior measures used in the Adolescent Health Survey and similar to those used in Project Eating Among Teens [24,25].

### Covariates

Demographic characteristics such as age, race/ethnicity, and sex were based on self-report. Adolescent household socioeconomic status was a composite based on the highest reported parental education, parental income, parental job status, and the number of social welfare benefits received [26]. Adolescents from higher income backgrounds may express more equitable gender attitudes [27]. Weight and height were measured by the interviewer at the end of the Wave II (1996) interview; body mass index (BMI) was calculated using the standard formula, weight (kilograms) divided by height (meters) squared (BMI = weight/height^2^). BMI was then converted into sex- and age-specific percentiles in accordance with guidelines from the U.S. Centers for Disease Control and Prevention [28].

### Statistical analysis

Data analysis was performed in 2018–2019 using R (RStudio, Boston, MA). We used logistic regression adjusting for survey weights and clustering on school groupings using the survey package in R to test the associations between baseline gender normativity score (at the individual and school levels) and the outcomes of weight control attempts or behaviors at 1- and 7-year follow-up. All models were adjusted for age, race/ethnicity, socioeconomic status, BMI percentile, and weight control attempts or behaviors at baseline [[29], [30], [31]]. Loess plots were created to show a smooth line to easily visualize the trends in the relationships between significant associations between predictors and outcomes identified in the regression models using locally weighted smoothing on scatter plots. All analyses were disaggregated by sex as gender norms can have differing health consequences for girls and boys [17,23]. Alpha was set at 95% confidence interval, and *p* < .05 is considered significant.

## Results

Overall, 9,861 adolescents (5,151 girls and 4,710 boys) were included in the sample. Descriptive and demographic characteristics of the sample at baseline are shown in Table 1. Mean age was 16 years. The sample was racially and ethnically diverse, with 21% black/African American, 16% Hispanic/Latino, and 9% Asian/Pacific Islander. The mean gender normativity score was .69 (range .001–.999) for individuals and .69 (range .494–.836) for schools. Among adolescent girls at 1-year follow-up, 45% reported trying to lose weight, whereas 7% reported trying to gain weight. In contrast, among adolescent boys at 1-year follow-up, 30% reported trying to gain weight, whereas 22% reported trying to lose weight. Weight loss and weight gain attempts remained relatively stable at 7-year follow-up.

**Table 1:** Demographic and health characteristics of 12,441 adolescent participants in the National Longitudinal Study of Adolescent Health, stratified by gender

	Total	Female	Male
N	9,861	5,151	4,701
Demographic characteristics	Mean (SE)/%	Mean (SE)/%	Mean (SE)/%
Age, baseline (years)	15.8 (1.6)	15.7 (1.5)	15.9 (1.6)
Race/ethnicity^a^			
White (non-Hispanic)	63.2%	62.8%	64.0%
Black/African American (non-Hispanic)	21.0%	22.4%	19.9%
Hispanic/Latino	16.0%	15.3%	16.7%
Asian/Pacific Islander (non-Hispanic)	9.0%	8.8%	9.3%
Socioeconomic status, baseline^b^	.09 (1.32)	.07 (1.33)	.11 (1.31)
Body mass index percentile, follow-up	60.3 (28.2)	58.5 (27.7)	62.2 (28.6)
Gender normativity scores			
Gender normativity score (individual), baseline	.69 (.24)	.70 (.24)	.69 (.24)
Gender normativity score (school level), baseline	.69 (.06)	.70 (.06)	.69 (.05)
1-year follow-up outcomes (Wave II)			
Weight control attempts			
Trying to lose weight	33.7%	45.3%	21.6%
Trying to gain weight	18.6%	7.5%	30.2%
Not trying to change weight	47.7%	47.2%	48.1%
Weight control behaviors			
Weight loss behavior	14.9%	26.4%	4.4%
Weight gain or muscle-building behavior	15.4%	7.4%	22.7%
7-year follow-up outcomes (Wave III)			
Weight control attempts			
Trying to lose weight	33.6%	44.2%	22.5%
Trying to gain weight	16.4%	5.1%	28.1%
Not trying to change weight	49.9%	49.3%	50.6%
Weight control behaviors			
Weight loss behavior^c^	25.6%	34.7%	16.0%
Weight gain or muscle-building behavior^d^	16.3%	5.1%	27.9%

SE = standard error.

a Participants could select multiple race/ethnicities.

b Socioeconomic status was a composite based on the highest reported parental education, parental income, parental job status, and the number of social welfare benefits received.

c Weight loss behaviors include dieting, vomiting, fasting/skipping meals, or laxative/diuretic use to lose weight.

d Weight gain behaviors include eating more, eating different foods than normal, and taking food supplements to gain weight.

Associations between baseline gender normativity score and subsequent weight control attempts at 1-year follow-up are shown in Table 2. A higher baseline individual gender normativity score (i.e., more feminine for girls and more masculine for boys) was associated with weight loss attempts in girls (β = .10; *p* = .01) but was protective of weight loss attempts in boys (β = −.17; *p* < .001). A higher baseline individual gender normativity score was associated with weight gain attempts in boys (β = .18; *p* < .001) but not in girls. A higher baseline individual gender normativity score was protective of weight loss attempts in girls (β = −.10; *p* < .001) but not in boys.

**Table 2:** Associations between baseline gender normativity score and weight control attempts and behaviors at 1-year follow-up in the National Longitudinal Study of Adolescent to Adult Health

	Female			Male		
	Beta coefficient^a^	SE	*p*	Beta coefficient^a^	SE	*p*
Weight control attempts						
Not trying to change weight						
Gender normativity score (school level), baseline	.01	.05	.84	−.03	.04	.49
Gender normativity score (individual), baseline	−**.10**	**.03**	**.00**	−.02	.04	.55
Weight loss attempt						
Gender normativity score (school level), baseline	.00	.06	.99	.09	.07	.18
Gender normativity score (individual), baseline	**.10**	**.03**	**.01**	−**.17**	**.05**	**.00**
Weight gain attempt						
Gender normativity score (school level), baseline	.01	.07	.91	−.02	.05	.71
Gender normativity score (individual), baseline	.00	.06	.95	**.18**	**.05**	**.00**
Weight control behaviors						
Weight loss behavior^b^						
Gender normativity score (school level), baseline	.01	.05	.92	.01	.08	.92
Gender normativity score (individual), baseline	**.18**	**.04**	**.00**	**−.20**	**.07**	**.00**
Weight gain or muscle-building behavior^c^						
Gender normativity score (school level), baseline	.06	.11	.56	.04	.05	.44
Gender normativity score (individual), baseline	.03	.08	.76	**.16**	**.05**	**.00**

Bold indicates *p* < .05.

SE = standard error.

a Adjusted for age, race/ethnicity, socioeconomic status, body mass index percentile, and baseline weight control attempts or behaviors.

b Weight loss behaviors include dieting, vomiting, fasting/skipping meals, or laxative/diuretic use to lose weight.

c Weight gain behaviors include eating more, eating different foods than normal, and taking food supplements to gain weight.

Among those who reported weight control attempts, associations between baseline gender normativity score and subsequent weight control behaviors are shown in Table 2. A higher individual gender normativity score was associated with weight loss behaviors in girls (β = .18; *p* < .001) but was protective of weight loss behaviors in boys (β = −.20; *p* < .001). Higher baseline individual gender normativity was associated with weight gain behavior in boys (β = .16; *p* < .001), but not in girls.

Associations between baseline gender normativity scores and subsequent weight control attempts and behaviors at 7-year follow-up are shown in Table 3. A higher baseline individual gender normativity score was protective of weight loss attempts (β = −.15; *p* < .001) and weight loss behaviors (β = −.17; *p* < .001) in males at 7-year follow-up.

**Table 3:** Associations between baseline gender normativity score and weight control attempts and behaviors at 7-year follow-up in the National Longitudinal Study of Adolescent to Adult Health

	Female			Male		
	Beta coefficient^a^	SE	*p*	Beta coefficient^a^	SE	*p*
Weight control attempts						
Not trying to change weight						
Gender normativity score (school level), baseline	.06	.04	.15	.02	.04	.61
Gender normativity score (individual), baseline	−.01	.03	.81	−.05	.03	.10
Weight loss attempt						
Gender normativity score (school level), baseline	.02	.05	.70	−.01	.05	.83
Gender normativity score (individual), baseline	−.01	.03	.77	**−.15**	**.04**	**.00**
Weight gain attempt						
Gender normativity score (school level), baseline	.23	.12	.05	.03	.05	.47
Gender normativity score (individual), baseline	−.01	.09	.88	.06	.04	.12
Weight control behaviors						
Weight loss behavior^b^						
Gender normativity score (school level), baseline	−.04	.06	.53	.05	.05	.33
Gender normativity score (individual), baseline	.05	.03	.14	**−.17**	**.04**	**.00**
Weight gain or muscle-building behavior^c^						
Gender normativity score (school level), baseline	.23	.12	.05	.04	.05	.36
Gender normativity score (individual), baseline	.01	.09	.95	.07	.04	.09

Bold indicates *p* < .05.

SE = standard error.

a Adjusted for age, race/ethnicity, socioeconomic status, body mass index percentile, and baseline weight control attempts or behaviors.

b Weight loss behaviors include dieting, vomiting, fasting/skipping meals, or laxative/diuretic use to lose weight.

c Weight gain behaviors include eating more, eating different foods than normal, and taking food supplements to gain weight.

**Figure 1:**
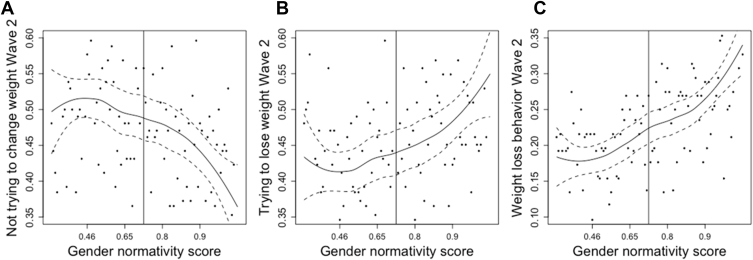
Loess plots showing significant associations between baseline gender normativity score and weight control attempts and behaviors in females.

**Figure 2:**
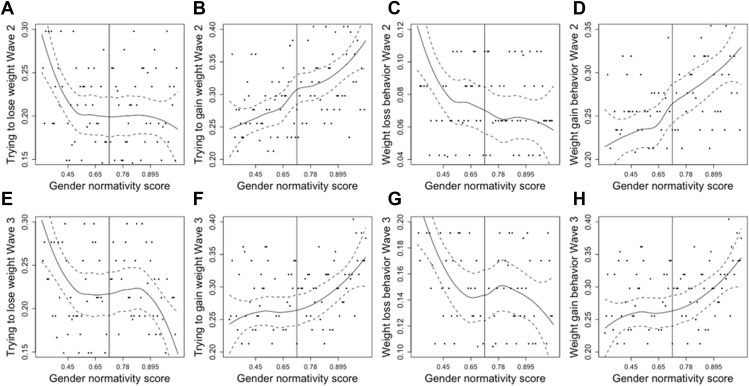
Loess plots showing significant associations between baseline gender normativity score and weight control attempts and behaviors in males.

The significant associations between baseline gender normativity score and weight control attempts and behaviors in girls (Figure 1) and boys (Figure 2) using loess plots are shown. Loess plots use locally weighted smoothing on scatter plots to show a smooth line to easily visualize the trends in a relationship between a predictor of interest and an outcome. In girls, the relationships between gender normativity scores and weight loss attempts (Figure 1B) and behaviors (Figure 1C) were slightly U-shaped. For those below the median in feminine norms, there was little association between norms and weight loss attempts, whereas for those above the median on feminine norms, as femininity increased, the likelihood of weight loss attempts increased. We see the same pattern for not wanting to change weight (Figure 1A). The negative association between gender normativity and not wanting to change weight was most prominent for those above the median on femininity. For men, although we see that the association between gender normativity and weight gain attempts (Figure 2B) and behaviors (Figure 2D) is fairly linear, the association between weight loss attempts (Figure 2A) and behaviors (Figure 2C) at Wave II seems to be strongest for those who are least masculine. For men who are less masculine, an increase in gender normativity is more strongly associated with weight loss attempts and behaviors than it is for those on the more masculine end of the gender normativity scale. In Wave III (Figure 2E–H), those associations seem to be negligible for those in the middle of the gender normativity scale, with stronger associations at both ends.

## Discussion

We find that adolescent gender norms—feminine behaviors in girls and masculine behaviors in boys—were positively associated with weight loss attempts and behaviors in girls but were negatively associated with weight loss attempts and behaviors in boys at 1-year follow-up. In boys, adolescent gender norms were positively associated with weight gain attempts and behaviors at 1-year follow-up. Although we saw a similar pattern for Wave III, the associations between adolescent gender normativity and weight gain behaviors were mostly attenuated and no longer significant, although adolescent gender norms were still negatively associated with weight loss attempts and behaviors in young men at 7-year follow-up. Gender norms can reinforce the thinner body ideal for girls and the bigger body ideal for boys, potentially leading to detrimental behaviors and health outcomes.

These findings add to the literature on how gender norms can affect health outcomes by specifically examining the relationships between adolescent gender norms and subsequent weight control attempts and behaviors. Although early femininity theory of eating disorders posited that high levels of femininity are associated with eating disorder pathology [32], subsequent research has had mixed findings and was mainly limited to adult college-age [3] or clinical [33] samples. We add to this literature by examining a large, nationally representative sample of adolescents, including males, in the U.S. and demonstrate how gender norms can affect health behaviors and outcomes over time longitudinally. Although gender norms were significantly associated with weight control attempts and behaviors in the school context at 1-year follow-up, these associations were attenuated and mostly no longer significant at 7-year follow-up outside of the high school environment. Furthermore, although most research on gender norms and body image has focused on female gender norms and thinness body ideals, we also examine weight gain or muscle-building body image ideals and associated behaviors particularly in males.

Overall conformity to feminine gender norms has been previously shown to be related to disordered eating symptoms [2] and pathology [1]. The relationship between feminine gender norms and weight loss attempts and behaviors may be explained through several mechanisms. Mahalik et al. [1] developed the Conformity to Feminine Norms Inventory that included several appearance-related norms including “Thinness,” “Look Young,” “Be Physically Attractive,” “Be Virginal,” and “Be Sexy.” In particular, conformity to thinness-oriented feminine norms may be detrimental for body esteem of women [2]. Concerns about physical attractiveness are higher in women than in men [4]. One study found that overweight women had the highest levels of body dissatisfaction compared with average or underweight women or men [4]. Women considered “normal weight” had the same levels of dissatisfaction as overweight men [4]. Gender norms that are not specifically appearance related, such as modesty, have also been shown to be associated with body dissatisfaction [1]. Modesty that leads females to downplay accomplishments might contribute to feelings of inadequacy in self-image [1].

Since the Add Health data collection period of 1994–2002, weight loss attempts and behaviors have remained relatively stable among adolescent girls but have increased among adolescent boys [5,10]. Among both males and females in Add Health from 1994 to 2002, purging behaviors remained relatively stable while diet pill use increased; however, these may be reflective of age changes as the cohort transitioned from adolescence to young adulthood rather than changes in trends over time [34].

We find that adolescent gender norms were associated with weight gain or muscle building behaviors in U.S., primarily male, adolescents. Since 1994–2002, when these data were collected, studies have reported a similar prevalence of weight gain or muscle building attempts among males (30% in 2015) [6]. Other studies with more recent data have found that masculine gender norms were associated with greater muscle dissatisfaction among young men [35]. In clinical samples, male patients with muscle dysmorphic disorder have also been shown to have greater adherence to masculine gender norms [36].

This study has several limitations. First, questions were based on self-report, which may be subject to reporting bias. The time frame used for measuring weight control behaviors in Add Health was shorter (past 7 days) than is often asked in other measures of weight control behaviors such as the Eating Disorder Examination Questionnaire (past 28 days) [37]. Body image was not measured. BMI was not measured at baseline; thus, follow-up BMI was used as a covariate. Selection bias is possible as we excluded participants with missing follow-up data; comparisons of demographic characteristics of those included versus excluded are shown in Appendix A and indicate that participants with higher socioeconomic status and white race were more likely to be retained. Gender norms were not directly measured in Add Health; thus, the gender normativity measure is a proxy that has been developed and applied to represent gender norms [18] but may miss subtler aspects of gender norms in these contexts. The adolescent context of the 1990s may be different than the present; however, the cohort study design allowed for longitudinal analysis of the data. As recent research suggests that modern-day adolescents view gender as more fluid and less constrained than previous generations [38], future research will be necessary to understand these associations across generations. It is possible that in a less gender-restricted social context, adhering strongly to gender norms may incur stigma and increase risks in different ways. Despite these limitations, strengths included a large, nationally representative, longitudinal sample of U.S. adolescents.

This research may have several clinical and public health implications. Mental health providers have viewed gender roles as critical to their theoretical, empirical, and clinical work [1]. Understanding gender norms and contextual pressures can assist health care and mental health providers to navigate social expectations that accompany being a girl or boy [39]. Clinicians should consider integrating gendered perspectives into the assessment, diagnosis, and treatment for eating disorders or body image–related issues [35,39]. Furthermore, school-based and community-level interventions to address gender norms may be developed for adolescents.

In conclusion, adolescent gender norms were associated with weight loss attempts and behaviors in girls but protective of weight loss attempts and behaviors in boys in the school context. In boys, adolescent gender norms were associated with weight gain attempts and behaviors in the school context. By 7-year follow-up, the associations between adolescent gender norms and weight control attempts and behaviors were attenuated. Gender norms may reinforce a thinner body ideal for girls and a larger, more muscular body ideal for boys, which are associated with differential health behaviors and outcomes.

## References

[1] Mahalik J.R., Morray E.B., Coonerty-Femiano A. (2005). Development of the conformity to feminine norms inventory. Sex Roles.

[2] Green M.A., Davids C.M., Skaggs A.K. (2008). Femininity and eating disorders. Eat Disord.

[3] Timko C., Striegel-Moore R., Silberstein L.R. (1987). Feminity/masculinity and disordered eating in women: How are they related?. Int J Eat Disord.

[4] Demarest J., Langer E. (1996). Perception of body shape by underweight average, and overweight men and women. Percept Mot Skills.

[5] Demissie Z., Lowry R., Eaton D.K. (2015). Trends in weight management goals and behaviors among 9th-12th grade students: United States, 1999-2009. Matern Child Health J.

[6] Nagata J.M., Bibbins-Domingo K., Garber A.K. (2019). Boys, bulk, and body ideals: Sex differences in weight gain attempts among adolescents in the United States. J Adolesc Health.

[7] Pope H.G., Olivardia R., Gruber A. (1999). Evolving ideals of male body image as seen through action toys. Int J Eat Disord.

[8] Nagata J.M., Murray S.B., Bibbins-Domingo K. (2019). Predictors of muscularity-oriented disordered eating in U.S. young adults: A prospective cohort study. Int J Eat Disord.

[9] Nagata J.M., Garber A.K., Tabler J. (2018). Prevalence and correlates of disordered eating behaviors among young adults with overweight or obesity. J Gen Intern Med.

[10] Chin S.N.M., Laverty A.A., Filippidis F.T. (2018). Trends and correlates of unhealthy dieting behaviours among adolescents in the United States, 1999-2013. BMC Public Health.

[11] Striegel-Moore R.H., Bulik C.M. (2007). Risk factors for eating disorders. Am Psychol.

[12] McKnight Investigators (2003). Risk factors for the onset of eating disorders in adolescent girls: Results of the McKnight longitudinal risk factor study. Am J Psychiatry.

[13] Neumark-Sztainer D., Wall M., Larson N.I. (2011). Dieting and disordered eating behaviors from adolescence to young adulthood: Findings from a 10-year longitudinal study. J Am Diet Assoc.

[14] French S.A., Story M., Downes B. (1995). Frequent dieting among adolescents: Psychosocial and health behavior correlates. Am J Public Health.

[15] Eisenberg M.E., Wall M., Neumark-Sztainer D. (2012). Muscle-enhancing behaviors among adolescent girls and boys. Pediatrics.

[16] Volpe U., Tortorella A., Manchia M. (2016). Eating disorders: What age at onset?. Psychiatry Res.

[17] Weber A.M., Cislaghi B., Meausoone V. (2019). How gender norms shape health: Insights from global survey data. Lancet.

[18] Fleming P.J., Harris K.M., Halpern C.T. (2017). Description and evaluation of a measurement technique for assessment of performing gender. Sex Roles.

[19] Nagata J.M., Garber A.K., Tabler J.L. (2018). Differential risk factors for unhealthy weight control behaviors by sex and weight status among U.S. adolescents. J Adolesc Health.

[20] Harris K.M., Halpern C.T., Whitsel E. The national longitudinal study of adolescent to adult health: Research design [online]. https://www.cpc.unc.edu/projects/addhealth/design/researchdesign_3618_regular.pdf.

[21] Cleveland H.H., Udry J.R., Chantala K. (2001). Environmental and genetic influences on sex-typed behaviors and attitudes of male and female adolescents. Person Soc Psychol Bull.

[22] Nowotny K.M., Peterson R.L., Boardman J.D. (2015). Gendered contexts: Variation in suicidal ideation by female and male youth across U.S. States. J Health Soc Behav.

[23] Shakya H.B., Domingue B.W., Nagata J.M. (2019). Adolescent gender norms and adult health outcomes in the US: A prospective cohort study. Lancet Child Adolesc Health.

[24] Neumark-Sztainer D., Story M., Resnick M.D. (1998). Lessons learned about adolescent nutrition from the Minnesota adolescent health survey. J Am Diet Assoc.

[25] Neumark-Sztainer D. Project EAT 2010 and F-EAT surveys - Derived variables and scales [online]. http://docs.sph.umn.edu/epich/eat/EAT2010_FEAT_Psychometrics.pdf.

[26] Belsky D.W., Domingue B.W., Wedow R. (2018). Genetic analysis of social-class mobility in five longitudinal studies. Proc Natl Acad Sci U S A.

[27] Kågesten A., Gibbs S., Blum R.W. (2016). Understanding factors that shape gender attitudes in early adolescence globally: A mixed-methods systematic review. PLoS One.

[28] Centers for Disease Control (2017). Defining adult overweight and obesity. https://www.cdc.gov/obesity/adult/defining.html.

[29] Neumark-Sztainer D., Wall M.M., Haines J.I. (2007). Shared risk and protective factors for overweight and disordered eating in adolescents. Am J Prev Med.

[30] Haines J., Kleinman K.P., Rifas-Shiman S.L. (2010). Examination of shared risk and protective factors for overweight and disordered eating among adolescents. Arch Pediatr Adolesc Med.

[31] Tabler J., Utz R.L. (2015). The influence of adolescent eating disorders or disordered eating behaviors on socioeconomic achievement in early adulthood. Int J Eat Disord.

[32] Boskind-Lodahl M. (1976). Cinderella's stepsisters: A feminist perspective on anorexia nervosa and bulimia. Signs.

[33] Srikameswaran S., Leichner P., Harper D. (1984). Sex role ideology among women with anorexia nervosa and bulimia. Int J Eat Disord.

[34] Stephen E.M., Rose J.S., Kenney L. (2014). Prevalence and correlates of unhealthy weight control behaviors: Findings from the national longitudinal study of adolescent health. J Eat Disord.

[35] Griffiths S., Murray S.B., Touyz S. (2015). Extending the masculinity hypothesis: An investigation of gender role conformity, body dissatisfaction, and disordered eating in young heterosexual men. Psychol Men Masculinities.

[36] Murray S.B., Rieger E., Karlov L. (2013). Masculinity and femininity in the divergence of male body image concerns. J Eat Disord.

[37] Fairburn C.G., Beglin S., Fairburn C.G. (2008). Eating disorder examination questionnaire. Cognitive Behavior Therapy and Eating Disorders.

[38] Gosling J. (2018). Gender fluidity reflected in contemporary society. Jung J.

[39] Steinfeldt J.A., Zakrajsek R., Carter H. (2011). Conformity to gender norms among female student-athletes: Implications for body image. Psychol Men Masculinity.

