# Supplementary material for: Gender Norms and Weight Control Behaviors in U.S. Adolescents: A Prospective Cohort Study (1994–2002)
Source: J Adolesc Health. 2020 Jan;66(1 Suppl):S34–41. doi: 10.1016/j.jadohealth.2019.08.020 (PMC6928570; doi:10.1016/j.jadohealth.2019.08.020)
Supplement: Appendix A [file mmc1.pdf]

Appendix A. Comparison of characteristics of participants in the National Longitudinal Study of Adolescent to Adult Health at baseline who were included versus excluded[a]

	Wave I only (n=18,922)	Wave I-III (n=9,861)
	Mean (SD) / %[b]	Mean (SD) / %[b]
White (non-Hispanic)	59.4%	63.0%
Black/African American (non-Hispanic)	24.0%	22.0%
Hispanic/Latino	18.0%	16.0%
Asian/Pacific Islander (non-Hispanic)	8.0%	8.0%
Age, years	16.6 (1.73)	15.8 (1.60)
Socio-economic status	-0.03 (1.33)	0.06 (1.34)

[a] Excluded due to missing one- or seven-year follow-up data, national sample weighting not incorporated into these analyses

[b] SD = standard deviation
